# Inhibition of *N*-Acetyltransferase 10 Suppresses the Progression of Prostate Cancer through Regulation of DNA Replication

**DOI:** 10.3390/ijms23126573

**Published:** 2022-06-12

**Authors:** Ningning Ma, Haijing Liu, Yaqian Wu, Mengfei Yao, Bo Zhang

**Affiliations:** Department of Pathology, School of Basic Medical Sciences, Peking University Health Science Center, 38 Xueyuan Road, Haidian District, Beijing 100191, China; maningning@bjmu.edu.cn (N.M.); liuhaijing@bjmu.edu.cn (H.L.); wuyaqiane@bjmu.edu.cn (Y.W.); yaomenfei119@sina.com (M.Y.)

**Keywords:** *N*-acetyltransferase 10, DNA replication, prostate cancer, CRPC, progression

## Abstract

Cancer suppression through the inhibition of *N*-acetyltransferase 10 (NAT10) by its specific inhibitor Remodelin has been demonstrated in a variety of human cancers. Here, we report the inhibitory effects of Remodelin on prostate cancer (PCa) cells and the possible associated mechanisms. The prostate cancer cell lines VCaP, LNCaP, PC3, and DU145 were used. The in vitro proliferation, migration, and invasion of cells were measured by a cell proliferation assay, colony formation, wound healing, and Transwell assays, respectively. In vivo tumor growth was analyzed by transplantation into nude mice. The inhibition of NAT10 by Remodelin not only suppressed growth, migration, and invasion in vitro, but also the in vivo cancer growth of prostate cancer cells. The involvement of NAT10 in DNA replication was assessed by EdU labeling, DNA spreading, iPOND, and ChIP-PCR assays. The inhibition of NAT10 by Remodelin slowed DNA replication. NAT10 was detected in the prereplication complex, and it could also bind to DNA replication origins. Furthermore, the interaction between NAT10 and CDC6 was analyzed by Co-IP. The altered expression of NAT10 was measured by immunofluorescence staining and Western blotting. Remodelin markedly reduced the levels of CDC6 and AR. The expression of NAT10 could be altered under either castration or noncastration conditions, and Remodelin still suppressed the growth of in vitro-induced castration-resistant prostate cancers. The analysis of a TCGA database revealed that the overexpression of NAT10, CDC6, and MCM7 in prostate cancers were correlated with the Gleason score and node metastasis. Our data demonstrated that Remodelin, an inhibitor of NAT10, effectively inhibits the growth of prostate cancer cells under either no castration or castration conditions, likely by impairing DNA replication.

## 1. Introduction

Prostate cancer (PCa) is currently the most common cancer in elderly men [1]. The prostate is an organ that is regulated by sex hormones, and so most prostate cancers are also regulated by androgen activity, which is hormone-dependent (castration sensitive). However, after a period of treatment with androgen antagonists (or castration), most prostate cancers become hormone-independent, which is known as castration-resistant prostate cancer (CRPC), with an increase in relapse and metastasis, resulting in fatal consequences for patients [2]. Alternative AR (androgen receptor) signaling is a characteristic of CRPC, and a vast number of investigations on AR amplification, variant expression, and the activation of signal transducers have been documented, but the critical mechanism remains to be elucidated. Upon binding to ligands, AR translocates to the nucleus to activate its target genes to promote cell proliferation, acting as a transcription factor, while in CRPC, ligand-independent mechanisms can also activate AR signaling, such as AR amplification and splice variant expression [3,4]. However, a non-transcriptional function of AR has also been discovered, in which AR can directly participate in the initiation of DNA replication by interacting with CDC6. In fact, Casodex, a nonsteroidal antiandrogen drug, represses the interaction between AR and CDC6, thereby inhibiting DNA replication. In recent years, DNA replication-related damage, primarily replication stress, has been clarified as an important mechanism underlying the genomic instability of cancer progression, and has been accepted as a hallmark of cancer [5,6]. Nevertheless, understanding regarding the role of AR-associated DNA replication in the development of CRPC is still limited.

NAT10 is a nucleolar protein that contains acetyltransferase and tRNA binding domains, and it mediates the acetylation of many types of molecules, such as histones, microtubules, tRNA, or mRNA [7,8,9]. In addition, NAT10 participates in mitotic cytokinesis and the cell stress response, and it is thus usually highly expressed in a variety of human cancers [9,10,11].

Remodelin, an inhibitor of NAT10 that is primarily used to relieve nuclear lamina defect-induced phenotypes, has been demonstrated to suppress a variety of human cancer cells, inhibiting their growth and cell cycle progression [12,13,14]. Our recent investigation further revealed that the inhibitory effects of Remodelin rely on the functional activity of NAT10 during DNA replication [15]. Although the role of the NAT10 inhibitor Remodelin in various human cancer cells has been explored in recent years, research that is related to prostate cancer is still lacking. In particular, the relationship between NAT10 and DNA replication suggests that NAT10 may be involved in the regulation of the biological activity of prostate cancer cells. Moreover, the fact that AR and CDC6-mediated DNA replication play a role in prostate cancers and their interaction alters the development of CRPC raises the potential for the utility of Remodelin in targeting DNA replication as a therapeutic approach in the treatment of prostate cancers.

In this study, we demonstrated the inhibitory effects of Remodelin on AR-positive and AR-negative PCas, revealing that NAT10 is involved in DNA replication, possibly through its interaction with CDC6 and AR, and we further clarified that Remodelin retains the ability to inhibit the growth of castration-resistant prostate cancer cells in vitro.

## 2. Results

### 2.1. Inhibition of NAT10 Suppresses the Growth of Both AR-Positive and AR-Negative Prostate Cancer Cells

**Figure 1:**
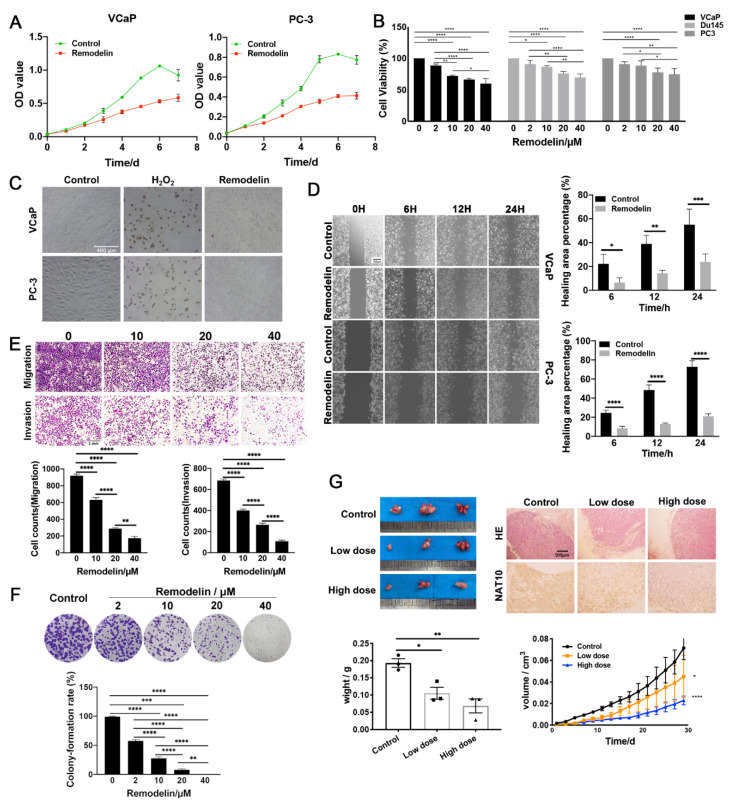
Inhibition of NAT10 suppresses the growth of both AR-positive and AR-negative prostate cancer cells. (**A**) Inhibition of NAT10 decreased the proliferation of prostate cancer cells in vitro. PC-3 and VCaP cells were treated with DMSO or Remodelin (20 µM), cell proliferation activity was measured every day, and a growth curve was drawn after 7 days. The experiment was repeated three times. Data are expressed as the mean ± SD. (**B**) PC-3, DU145, and VCaP cells were treated with different concentrations of Remodelin (0, 2, 10, 20, or 40 µM) for 48 h, and proliferation was evaluated. The level of statistical significance was compared using one-way ANOVA with Tukey post-hoc test to correct for multiple testing. (**C**) The analysis of cellular apoptosis in prostate cancer cells. VCaP and PC-3 cells were treated with either 20 μM Remodelin or 0.4 mM H_2_O_2_ for 24 h, respectively, and TUNEL assay was carried out according to the manufacturer’s instructions, as described in the Materials and Methods. (**D**,**E**) Inhibition of NAT10 decreased the migration and invasion of prostate cancer cells in vitro. (**D**) Cultured VCaP and PC-3 cells were subjected to wound healing assays with DMSO or Remodelin (20 µM). The level of statistical significance was compared using a t-test. *p* value versus control group. (**E**) VCaP cells were pretreated with different concentrations of Remodelin (0, 10, 20, or 40 µM) for 24 h, followed by Transwell assay. Representative images in each group are shown, and the data were quantified from triplicate experiments, and are presented as the mean ± SD. The level of statistical significance was compared using one-way ANOVA with Tukey post-hoc test to correct for multiple testing. (**F**) VCaP cells were treated with Remodelin (2, 10, 20, or 40 μM) at different concentrations, and the DMSO treatment group was used as the control group. Two weeks later, the colonies were stained, and the colony formation rate was statistically analyzed. The level of statistical significance was compared using one-way ANOVA with Tukey post-hoc test. (**G**) PC-3 cells were used in nude mouse transplantation experiments. One week after subcutaneous inoculation of PC-3 cells into the armpits of male nude mice, tumor size was measured every two days and the growth curve of the tumor volume was drawn. One-way ANOVA with Tukey post-hoc test and Dunnett post-hoc test. Tumors excised from euthanized mice were weighed, imaged, stained with HE, and compared using *t*-tests. Representative images in each group are shown, and the data were quantified from triplicate experiments and are presented as the mean ± SD. The level of statistical significance was *, *p* < 0.05; **, *p* < 0.01; ***, *p* < 0.001; ****, *p* < 0.0001, ns, no significance.

Previous studies have shown that using CRISPR/Cas9 technology to completely knock out NAT10 in colorectal cancer cells can lead to cell death, demonstrating that NAT10 is essential for the proliferation of colorectal cancer cells [15]. To further confirm the effects of NAT10 activity on the proliferation of prostate cancer cells, AR-positive VCaP and AR-negative PC-3 cells were treated with Remodelin and subsequently evaluated using the MTS method. Both types of cells treated with Remodelin displayed significantly decreased cell proliferation activity over time, compared to the control group (Figure 1A). The attenuation of proliferation by Remodelin was dose-dependent, indicating that the degree of NAT10 activity was closely related to the proliferation of prostate cancer cells (Figure 1B). Moreover, Remodelin-treated cells were subjected to a TUNEL assay for evaluating apoptosis, and the results showed that few cells were apoptotic-positive in the presence of Remodelin (20 μM), but that hydrogen peroxide (H_2_O_2_) could induce marked amounts of apoptosis (Figure 1C).

In addition, cell migration was assessed by wound healing experiments to observe whether it was affected by the expression of NAT10. The results showed that in both the AR-positive VCaP cell line and the androgen receptor-negative PC-3 cell line, the wound healing ability of the Remodelin treatment group was weaker than that of the control group (Figure 1D). Moreover, the Remodelin treatment group also exhibited decreased migration and invasion in a dose-dependent manner (Figure 1E).

Furthermore, colony formation experiments were conducted to study the inhibitory effect of different doses of Remodelin on VCaP cell proliferation. The experimental results revealed that colony formation ability decreased significantly with increasing Remodelin concentration (Figure 1F).

To further clarify the effects of NAT10 activity on the progression of prostate cancer in vivo, PC-3 cells were used in nude mouse transplantation experiments. One week after subcutaneous inoculation of PC-3 cells into the armpits of male nude mice, low-dose (2 mg/kg) and high-dose (20 mg/kg) Remodelin were injected every two days for 4 weeks. The data revealed that Remodelin significantly reduced AR-negative prostate cancer tumor growth, and in the high-dose Remodelin group, xenograft tumor weight at the endpoint was also much smaller than that in the low-dose group (Figure 1G).

These results suggested that the NAT10 inhibitor suppresses the growth and tumorigenesis potential of prostate cancer cells, both in vitro and in vivo.

### 2.2. Inhibition of NAT10 Slows DNA Replication in Prostate Cancer Cells

**Figure 2:**
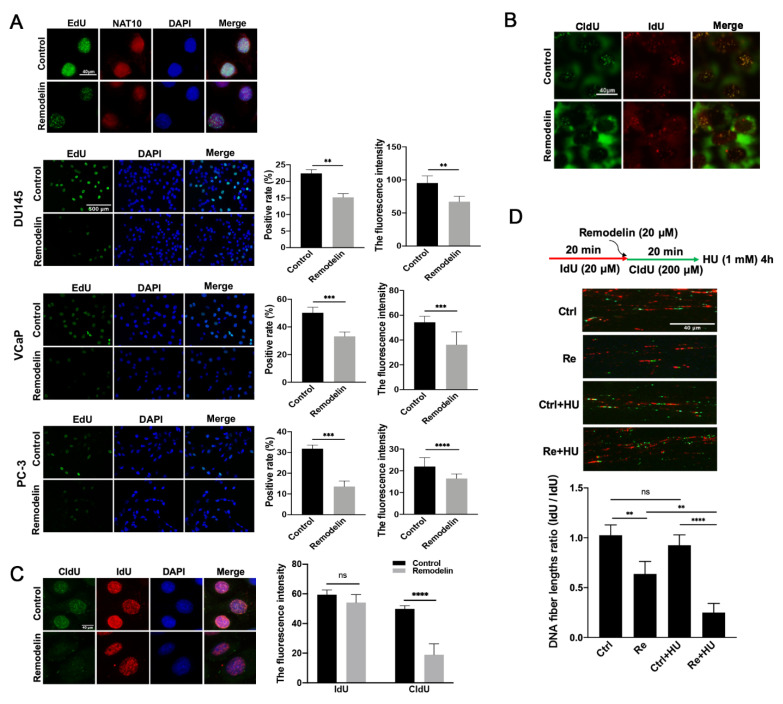
Inhibition of NAT10 slows DNA replication in prostate cancer cells. (**A**) In a variety of prostate cancer cells, Remodelin was used to inhibit the activity of NAT10, and then immunofluorescence co-staining was performed. NAT10 (red) and EdU (green) DAPI (blue) were compared with the control group, and differences in the EdU positive rate and the fluorescence intensity were analyzed. (**B**) Cells were pretreated with Remodelin (20 µM) for 24 h, treated with IdU (20 µM) for 20 min, and finally, treated with CldU (200 µM) for 60 min, and the cells were collected for immunofluorescence co-staining. (**C**) Cells were treated with IdU (20 μM) for 20 min, followed by Remodelin treatment for 4 h, with the DMSO treatment group as the control, and finally treated with CldU (200 μM) for 1 h. The cells were collected for immunofluorescence co-staining, IdU (red), CldU (green), DAPI (blue), and the fluorescence intensity were analyzed. *T*-test; **, *p* < 0.01; ***, *p* < 0.001; ****, *p* < 0.0001. (**D**) DNA fiber assay showing the influence of NAT10 on DNA replication forks. Control: IdU 20 min, CldU 20 min. Control + Hu: IdU 20 min, CldU 20 min, Hu (1 mM) 4 h. Remodelin: IdU 20 min, Remodelin + CldU 20 min, Remodelin + Hu: IdU 20 min, Remodelin + CldU 20 min, Hu 4 h, and the DNA fiber length ratios were analyzed. At least 500 cells were evaluated in each experiment. The representative images in each group are shown, and the data were quantified from triplicate experiments, and are presented as mean ± SD.

In previous studies, the genomic depletion or chemical inhibition of NAT10 was confirmed to decrease the growth, cell cycle progression and BrdU incorporation of colorectal cancer cells [15]. We speculated that Remodelin could also reduce the proliferation of prostate cancer cells by impairing DNA replication. EdU (5-ethynyl-2′-deoxyuridine) incorporated was used to label newly synthesized DNA in prostate cancer cell lines (VCaP, PC-3, DU145) in the presence or absence of Remodelin. The number of EdU-positive cells was quantified under fluorescence microscopy, and the fluorescence intensity was calculated. The results showed that compared to the control group, the Remodelin treatment groups of three cell lines showed a significant decrease in both the positive labeling rate and the fluorescence intensity (Figure 2A).

In addition, we used the thymidine analogs IdU and CldU to continuously label DNA replication sites. Cells were pretreated with Remodelin (20 μM) or DMSO (control) for 24 h, labeled with IdU (20 μM) for 20 min, and finally labeled with CldU (200 μM) for 60 min. After cell extraction, immunofluorescence staining was performed. A comparison between the Remodelin inhibition group and the control group revealed that both the staining foci of IdU and the staining foci of CldU were significantly reduced (Figure 2B). In order to show the Remodelin’s inhibitory effect on the replication sites more clearly, we applied Remodelin treatment between the IdU and CldU markers, followed by the fluorescent staining of cells. By comparing the Remodelin inhibitory group with the control group, We found that the IdU staining results were similar, while CldU staining foci were significantly reduced (Figure 2C). These results further confirmed that Remodelin treatment led to decreased DNA synthesis.

Moreover, the Remodelin-mediated inhibition of DNA replication was further confirmed by DNA fiber spreads. The extension length of DNA replication in the Remodelin treatment group was significantly shorter than that in the control group (Figure 2C). Furthermore, additional treatment with HU (hydroxyurea), an inhibitor of DNA synthesis that induces instability of the DNA replication fork [16,17], induced a significant reduction in DNA replication forks (Figure 2D). This finding shows that the inhibition of NAT10 by Remodelin reduces and destabilizes the replication forks.

Taken together, these results indicate that the inhibition of NAT10 slows DNA replication in PCa cells.

### 2.3. NAT10 Is Involved in DNA Replication

**Figure 3:**
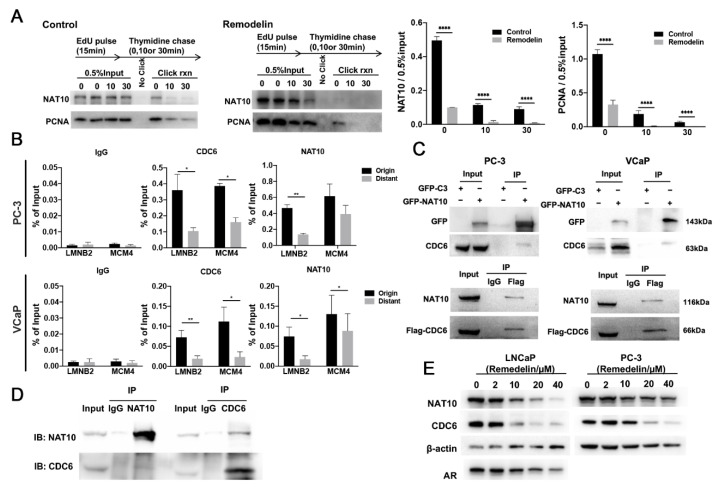
NAT10 is involved in the assembly of the DNA replication complex. (**A**) NAT10 binding to the nascent DNA. VCaP was pretreated with or without Remodelin for 12 h. After EdU (15 µM) labeling of DNA for 15 min, thymidine chases were used for 0, 10, and 30 min. The proteins cross-linked with DNA were separated using iPOND technology, and then Western blotting was used for detection, followed by statistical analysis. *T*-test; ****, *p* < 0.0001. (**B**) NAT10 is specifically distributed at the origin of the DNA replication site. The DNA fragments that bind to the NAT10 and CDC6 proteins in the VCaP and PC-3 cells were extracted as templates, with the known proximal and distal sequences of the two replication sites of LMNB2 and MCM4 used for primer design. Real-time quantitative PCR was performed, and the results were analyzed. *T*-test; *, *p* < 0.05; **, *p* < 0.01. (**C**,**D**) NAT10 interacts with CDC6. (**C**) VCaP and PC-3 cells were exogenously transfected with GFP-NAT10 or Flag-CDC6, and protein immunoprecipitation was performed. (**D**) Endogenous immunoprecipitation of VCaP cells with NAT10 antibody and CDC6 antibody. (**E**) Downregulation of NAT10 reduced the levels of AR and CDC6. After Remodelin gradient concentration (0, 2, 10, 20, or 40 µM) treatment, the levels of NAT10, AR and CDC6 in the PC-3 and LNCaP cells were measured by Western blotting, and statistics were performed. Representative images in each group are shown, and the data were quantified from triplicate experiments and are presented as the mean ± SD.

The fact that the inhibition of NAT10 slows DNA replication suggests that NAT10 could be directly involved in the initiation of DNA replication. An isolation of proteins on nascent DNA (iPOND) assay was performed to detect the existence of NAT10 on newly synthesized DNA [18] using an EdU pulse tracking experiment. First, the cells were incubated with EdU and then chased by adding thymidine. At this time, we monitored changes in chromatin at different distances from the replication fork, to determine how proteins that are related to the EdU-labeled DNA fragments varied with time and distance. Cell proliferating nuclear antigen (PCNA) is a global hub in DNA metabolism that interacts with a large number of proteins that are involved in a variety of DNA-related processes [19]. iPOND results showed that NAT10 gradually decreased with increasing thymidine incorporation time, showing the same trend as PCNA (Figure 3A), while the inhibition of NAT10 by Remodelin also reduced the activity of PCNA. These results confirm that NAT10 is part of the replisome, and they might suggest that Remodelin decreases the number of active replication forks or alternatively destabilizes ongoing replication forks.

To examine whether NAT10 is present at two well-known replication sites, co-immunoprecipitation of NAT10 and two well-defined origins near the lamin B2 (LMNB2) and MCM4 genes were analyzed using chromatin immunoprecipitation (ChIP). In the presence of anti-NAT10 antibodies, the abundance of proximal and distal sequences near the LMNB2 and MCM4 origins in immunoprecipitation from both VCaP and PC3 cells were measured using quantitative real-time PCR. The results showed that anti-NAT10 antibodies efficiently enriched the origin sequences LMNB2 and MCM4 (Figure 3B), and the enrichment of proximal sequences was significantly greater than that of the distal regions (Figure 3B). In contrast, the enrichment of the control IgG was below 0.005% of the inputs. At the same time, CDC6, a key factor in the initiation complex of DNA replication, was also measured by ChIP assay, and similar results were observed (Figure 3B). These data indicate that NAT10 does indeed bind to the replication origins of DNA.

Previous reports have revealed that both AR and CDC6 are involved in the initiation of DNA replication, and that the non-steroidal antiandrogen drug Casodex relieves the interaction between AR and CDC6, inhibiting DNA replication [20,21,22]. Therefore, the interaction between NAT10 and CDC6 was further investigated. The results showed that ectopically expressed GFP-NAT10 coprecipitated with endogenous CDC6, while the ectopic expression of Flag-CDC6 coprecipitated with endogenous NAT10 in VCaP and PC-3 cells (Figure 3C). In addition, endogenous CDC6 coprecipitated with endogenous NAT10, and vice versa (Figure 3D). These results confirmed the potential interaction between NAT10 and CDC6 in vivo.

Moreover, the inhibition of NAT10 by Remodelin reduced the levels of CDC6 in either AR-positive LNCaP or AR-negative PC-3 cells (Figure 3E) in a dose-dependent manner, indicating that the inhibition of NAT10 downregulates the expression of CDC6.

These results implied that NAT10 is anchored near the origin of the DNA replication site by its interaction with CDC6.

### 2.4. Expression of NAT10 Could Be Altered by Treatment with AR Modulators

**Figure 4:**
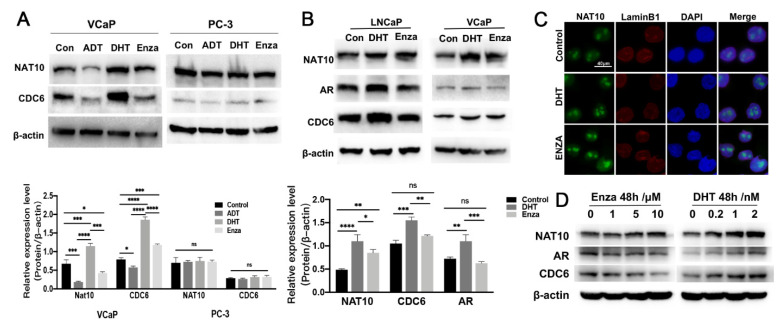
Altered expression of NAT10 in castration and non-castration of prostate cancer cells. (**A**) VCaP and PC-3 cells were treated with ADT, DHT (1 nM), and enzalutamide (10 µM) for 1 week; levels of NAT10 and CDC6 in VCaP and PC-3 cells were measured by Western blotting (upper panel), and statistics were performed (lower panel). One-way ANOVA with Tukey post-hoc test; *, *p* < 0.05; ***, *p* < 0.001; ****, *p* < 0.0001; ns, no significance. The experiment was repeated three times. (**B**,**C**) Both DHT and androgen receptor antagonists increased the expression levels of NAT10. VCaP or LNCaP cells were treated with DHT (1 nM) and enzalutamide (10 µM) for 48 h, and then subjected to Western blotting analysis (**B**) and immunofluorescence staining (**C**). (**B**) Levels of NAT10, AR, and CDC6 in VCaP and LNCaP cells were measured by Western blotting (upper panel), and statistics were performed (lower panel). One-way ANOVA with Tukey post-hoc test; *, *p* < 0.05; **, *p* < 0.01; ***, *p* < 0.001; ****, *p* < 0.0001; ns, no significance. (**C**) NAT10 (green), lamin B1 (red), and DAPI (blue). (**D**) DHT or enzalutamide gradient concentration treatment for 48 h. Levels of NAT10, AR, and CDC6 were measured in LNCaP cells.

Several previous reports have demonstrated that an interaction between CDC6 and AR is involved in the regulation of DNA replication initiation [21,22,23]. To determine whether the changes in NAT10 in the drug treatment of prostate cancer cells are related to AR, we used AR-negative PC-3 cells and AR-positive VCaP cells for comparison. VCaP and PC-3 cells were treated with androgen deprivation (ADT), dihydrotestosterone (DHT, 1 nM), or enzalutamide (Enza, 10 µM) for 1 week, and Western blotting analysis was performed (Figure 4A). In VCaP cells, the protein expression levels of NAT10 and CDC6 were downregulated under ADT treatment, but upregulated under DHT treatment, while PC3 cells exhibited no significant change in these proteins, indicating that the expression of both NAT10 and CDC6 is sensitive to AR signaling status.

To further explore the relationship between NAT10 expression and AR-positive prostate cancer cells, VCaP and LNCaP cells were treated with an AR agonist (DHT) or an AR antagonist (Enza) for 48 h, and protein levels of NAT10, AR, and CDC6 were analyzed by Western blotting. In the DHT-treated group, the expression of AR and CDC6 was upregulated compared with the control group, while the expression of AR and CDC6 in the Enza-treated group not significantly different to the control group. However, both the DHT- and Enza-treated groups exhibited an upregulation of NAT10 (Figure 4B). Immunofluorescence also showed that in the AR-positive VCaP cells, the fluorescence intensity of NAT10 was enhanced after DHT and Enza treatment (Figure 4C). In addition, further analysis demonstrated that the expression of NAT10 in VCaP cells was correlated with the concentration of DHT or enzalutamide treatment (Figure 4D) in a dose-dependent manner.

These results implied that expression of NAT10 is not only correlated with AR status, but is also altered in the presence of AR modulators.

### 2.5. Inhibition of NAT10 Suppresses Castration-Resistant Prostate Cancer Cells In Vitro

**Figure 5:**
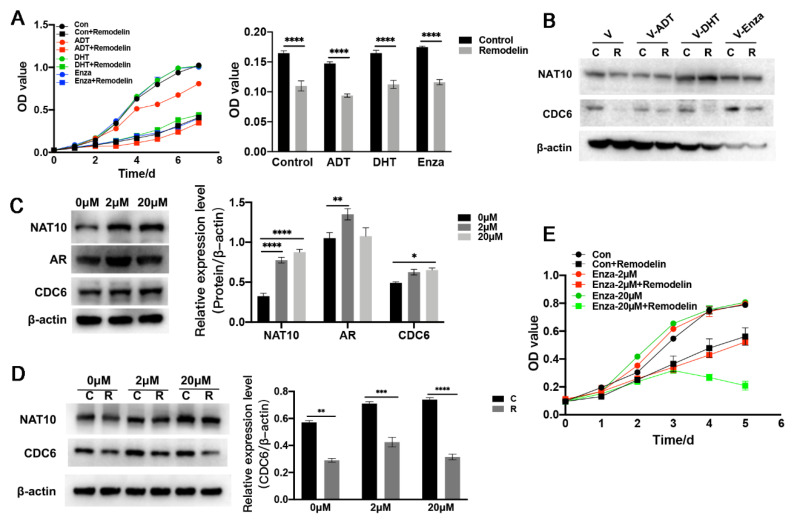
Inhibition of NAT10 suppresses castration-resistant prostate cancer cells in vitro. (**A**) Right panel: VCaP cells were treated with DMSO or Remodelin (20 µM), combined with cells pretreated with DHT, enzalutamide, and ADT, cell proliferation was evaluated every day, and a growth curve was created after 7 days. Left panel: VCaP cells were treated with DMSO or Remodelin (20 µM), combined with cells pretreated with DHT, enzalutamide, and ADT for 7 d, cell proliferation was assessed and analyzed by MTS assay. *T*-test; ****, *p* < 0.0001. (**B**) VCaP cells were treated with DMSO or Remodelin (20 µM), combined with cells pretreated with DHT, enzalutamide, and ADT for 24 h. Levels of NAT10 and CDC6 were measured by Western blotting. (**C**) VCaP cells were pretreated with enzalutamide for at least 4 weeks to generate enzalutamide-tolerant (2 μM) and enzalutamide-resistant (20 μM) cells. Levels of NAT10, AR, and CDC6 were measured by Western blotting (left panel), and statistical analysis was performed (right panel). One-way ANOVA with Tukey post-hoc test; *, *p* < 0.05; **, *p* < 0.01; ****, *p* < 0.0001. (**D**) Enzalutamide-tolerant (2 μM) and enzalutamide-resistant (20 μM) cells were treated with Remodelin or DMSO. Expression levels of NAT10 and CDC6 were determined by Western blotting and statistical analysis was performed (right panel). *T*-test; **, *p* < 0.01; ***, *p* < 0.001; ****, *p* < 0.0001. (**E**) Cell proliferation of enzalutamide-tolerant (2 μM) and enzalutamide-resistant (20 μM) cells was evaluated every day, and a growth curve was created after 5 days. Representative images in each group are shown, and the data were quantified from triplicate experiments, and are presented as the mean ± SD.

Considering the association of NAT10 expression with AR status and its alterations by AR modulators, there is a possibility that the activity of NAT10 could be involved in the castration treatment of prostate cancers. To further clarify whether NAT10 inhibition still affected castration-resistant prostate cancer cells, AR-positive VCaP cells were subjected to one week of short-term treatment with ADT, DHT, and enzalutamide, and the growth of these cells in the presence of Remodelin (20 µM) was analyzed. The results showed that the inhibition of NAT10 activity by Remodelin suppressed the growth of these cells under both conditions (Figure 5A). In addition, Western blotting showed that Remodelin reduced the expression of CDC6, but it slightly affected the expression of NAT10 in ADT-, DHT-, and Enza-treated cells (Figure 5B).

At the same time, to explore the effects of castration on prostate cancer, VCaP cells were subjected to long-term treatment with enzalutamide at either low (2 μM) or high (20 μM) concentrations for at least four weeks, and the expression of NAT10, CDC6, and AR was measured. Both enzalutamide tolerance (2 μM) and enzalutamide resistance (20 μM) exhibited an upregulated expression of NAT10 (Figure 5C). Interestingly, VCaP cells undergoing (de)androgen treatment and AR antagonist treatment exhibited effects on proliferation activity in response to NAT10 activity, and the expression of CDC6 changed accordingly (Figure 5C). Nevertheless, Remodelin still inhibited the expression of NAT10 and CDC6 (Figure 5D), indicating that the inhibition of NAT10 still suppresses the growth of VCaP cells in long-term castration treatment (Figure 5E).

These results suggest that the inhibition of NAT10 could suppress both short-term and long-term castration-treated AR-positive prostate cancer cells in vitro.

### 2.6. NAT10 Expression and its Correlation with DNA Replication Factors and Clinicopathological Features in Prostate Cancer

**Figure 6:**
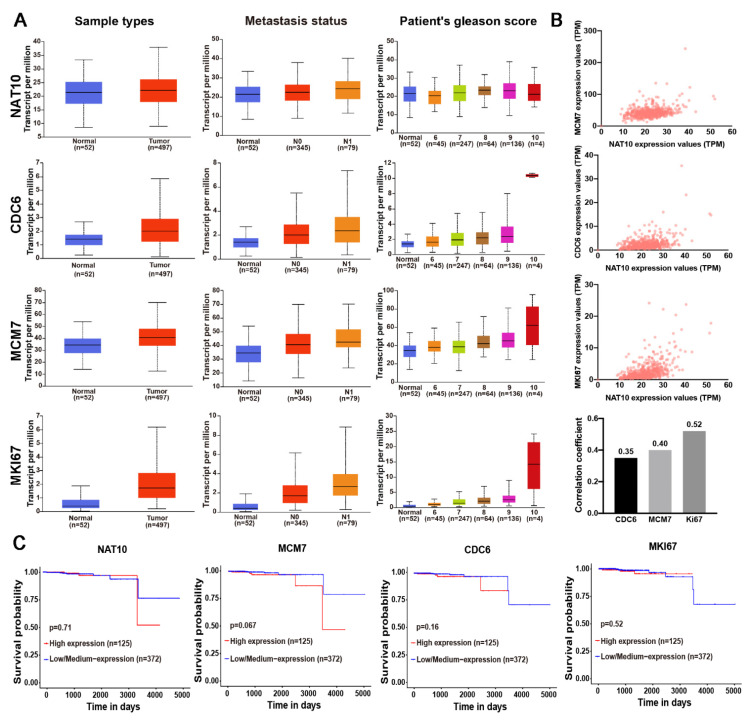
Correlations between NAT10 expression, and clinicopathological features of prostate cancers. (**A**) Box-whisker plots showing the expression of NAT10, CDC6, MCM7, and MKI67 in subgroups of prostate adenocarcinoma samples (PRAD). Left column, boxplot showing relative expression of protein in normal and PRAD samples. Middle column, boxplot showing relative expression of protein in normal and metastasis status. Right column, boxplot showing relative expression of protein in normal samples and samples with different Gleason scores. (**B**) Genes positively correlated with NAT10 in PRAD. Gene expression correlation between NAT10 and CDC6, and MCM7, MKI67 in prostate cancer (r ≥ 0.8, very strong correlation; 0.6–0.8, strong correlation; 0.4–0.6, moderate correlation; 0.2–0.4, weak correlation; <0.2, very weak correlation or no correlation). (**C**) Effect of protein expression level on PRAD patient survival. All data are from TCGA database and were analyzed using the UALCAN website.

NAT10 expression and its correlations with DNA replication factors and the clinicopathological features of prostate cancers were analyzed using prostate cancer data from the TCGA database on the UALCAN website (http://ualcan.path.uab.edu, accessed on 12 January 2022). The results revealed that expression levels of NAT10 and Ki67, CDC6, and MCM7 were increased in prostate cancers (Figure 6A). There were correlations to some extent between NAT10 and CDC6, and MCM7 and Ki67 (Figure 6B). In addition, NAT10 expression was correlated with node metastasis and a high Gleason score (Table 1). Similarly, CDC6, MCM7, and Ki67 expression was also correlated with node metastasis and high Gleason scores, and a high expression of MCM7 and Ki67 was particularly credited to the Gleason 10 score group (Table 1).

Expression of NAT10, CDC6, MCM7, and Ki67 was also correlated with clinical survival to varying extents (Figure 6C).

Analysis of a TCGA cohort revealed that the expression levels of NAT10 and Ki67, CDC6, and MCM7 were generally involved in the proliferation of prostate cancer cells and their differentiation (Gleason pattern), metastasis and clinical outcome, indicating their possible roles in promoting the progression of prostate cancers.

## 3. Discussion

The initiation of DNA replication has been extensively elucidated, revealing an extremely complicated process. The origin licensing/firing of DNA replication is initiated by the origin recognition complex (ORC), with CDC6 binding to the origin of replication and then recruiting a DNA helicase composed of the minichromosome maintenance (MCM) 2–7 proteins and Cdt1 at the replication start point, forming a pre-replicative complex (RC) to trigger start point authorization [24]. Surprisingly, an early report indicated that AR should be one of the permissive factors leading to the initiation of DNA replication in androgen-dependent PCa cells [25]. Further publications have extended this finding in detail, indicating that CDC6 gene expression is regulated by AR, in which AR binds to AR-response elements in the promoter of CDC6 and activates its transcription through either interaction with other transcriptional activators, or repressors or epigenetic modifications. Then, the activation of CDC6 expression by AR directly promotes cell cycle progression and the proliferation of AR-dependent PCa cells [21]. Moreover, another investigation revealed that as a component of the prereplication complex, AR directly interacts with CDC6, together with cyclins E and A and DNA polymerase α, as well as PCNA, to drive LNCaP cells from G1 phase to S phase [26]. A more recent study has shown that CDC6 is an AR target gene that is upregulated during PCa progression [22]. In addition, AR interacts with other initiation factors of DNA replication, such as MCM7 [27].

Our study revealed that NAT10 could bind to the DNA replication complex like CDC6 and have a direct interaction with CDC6, implying that NAT10 may directly participate in the pre-replication complex. At the same time, given that the inhibition of NAT10 by Remodelin reduced the levels of CDC6, the possibility that the interaction of NAT10 and CDC6 could be involved in the stability of proteins exists. In addition, the levels of NAT10 are closely related to AR status, and they were downregulated in ADT treatment, but markedly elevated in the presence of DHT, while the inhibition of NAT10 by Remodelin did not significantly affect AR status. It is possible that the expression of NAT10 is regulated by AR status. Therefore, the described investigation adds more evidence that AR regulates DNA replication, but these details still need further verification.

Interestingly, in response to enzalutamide treatment, AR and CDC6 were downregulated, but in contrast, NAT10 was upregulated, indicating a differential mechanism in the AR modulator. Our previous studies have shown that the expression of NAT10 is activated under cellular stress conditions, such as oxidative stress or DNA damage [10]. Studies have shown that CDC6 protects the integrity of the genome by activating the DDR, and that the knockdown of either AR or CDC6 induces replication-related DNA damage [22,28]. It can be assumed that NAT10 activation results from disrupting DNA replication. Future research on NAT10 in the presence of AR antagonists would be meaningful for the elucidation of CRPC.

Androgen deprivation therapy has been the standard therapy for advanced and metastatic prostate cancer. However, prostate cancers almost always evolve strategies to grow at low levels of androgen, and have even developed resistance to the second-generation AR antagonist enzalutamide, or the androgen biosynthesis inhibitor abiraterone acetate [29,30], a state known as castration-resistant prostate cancer (CRPC) [31]. A variety of investigations have demonstrated multiple escape pathways for the development of castration resistance [32,33]. Nevertheless, altered AR signaling has been identified to play a major role in advanced CRPC, including AR amplification and signaling crosstalk. The PSA doubling time has been verified as an indicator of prostate cancer, which usually precedes clinically detectable cancer relapse, suggesting the importance of proliferation dynamics in prostate cancer cells [34]. Actually, MCM7 amplification or the overexpression of CDC6 has been clarified in the progression of CRPC, reinforcing how DNA replication plays an important role in CRPC. In addition, the nonsteroidal antiandrogen drug Casodex, which is used to treat advanced prostate cancer, represses the interaction between AR and CDC6, inhibiting DNA replication [21]. In light of this finding, the targeting of NAT10 in PCa cells could be an effective approach for CRPC treatment. As revealed in our experiments, Remodelin inhibits the growth of prostate cancer cells in either short-term or long-term castration treatment.

Remodelin has been demonstrated to inhibit many kinds of human cancers and to delay cell cycle progression, but its mechanism is unclear. In our previous research, we revealed that either Remodelin treatment or the depletion of NAT10 inhibits the DNA replication of cancer cells, influencing the acetylation status of chromatin [15]. In the described investigation, we further demonstrated that NAT10 is directly involved in DNA replication, where NAT10 influences the complex assembly of DNA replication. Considering its acetylation activity, NAT10 could make the origin of DNA replication more accessible to CDC6 or other initiators by influencing the status of chromatin. In addition, the treatment of Remodelin could suppress the growth of cancer cells but not induce apoptosis, consistent with our previous observation that Remodelin has little cytotoxicity [35]. Remodelin can significantly inhibit the expression levels of CDC6 in prostate cancer cells and the proliferation ability of prostate cancer cells in vitro, regardless of whether the cells were treated with androgen-removed or androgen-treated cells (Figure 5A,B). When the CRPC cells induced in vitro were treated with Remodelin, Remodelin still showed a strong inhibitory effect on the expression level of CDC6 and the cell proliferation rate of cancer cells (Figure 5D,E). This also implied that the anti-neoplastic effects of Remodelin through NAT10 inhibition should be credited to the slowing down of DNA replication, which could consequently attenuate replication stress-associated genomic instability, and ultimately delay the progression of prostate cancer. It could be rational that androgen deprivation therapy should combine Remodelin administration to enforce the inhibition of DNA replication, and at the same time, delay the development of CRPC. In similar, for CRPC, Remodelin could also be integrated into systemic treatments in conjugation with chemotherapy or other current approaches. Nevertheless, more details regarding this process still need to be defined.

Although extensive investigation has been performed, predictable indicators of CRPC progression are now well-characterized. Given the importance of the proliferative potential in CRPC, the expression of DNA replication-related factors could be considered. CDC6, MCM7, and Ki67 were highly expressed in the high Gleason grading score in our analysis of the TCGA dataset (Figure 6). The detection of NAT10, Ki67, CDC6, or MCM7 levels could be helpful in clinical practice. Moreover, our research demonstrated that Remodelin, an inhibitor of NAT10, effectively inhibits the growth of PCa cells under both no-castration and castration conditions. In addition, our previous research demonstrated that Remodelin exerts no obvious cytotoxicity in vitro, and inhibits hypoxia-induced HIF expression [35]. Therefore, Remodelin could potentially be used in combination with radiotherapy, chemotherapy, and other therapeutic strategies for the clinical treatment of prostate cancers.

## 4. Materials and Methods

### 4.1. Plasmids and Reagents

CDC6 tagged with a C-terminal 3×FLAG tag (pcDNA3.1-3×Flag) was purchased from YouBio Biotechnology (Changsha, China). NAT10 tagged with a pEGFP-C3 tag and the rabbit polyclonal antibody against human NAT10 have been previously described [36]. Transient transfection was performed using Lipofectamine 2000 (Invitrogen, Carlsbad, CA, USA) according to the manufacturer’s recommendations. DAPI (C0060) was purchased from Solarbio (Beijing, China). Remodelin (S7641), dihydrotestosterone (DHT, S4757), and enzalutamide (S1250) were purchased from Selleck (Houston, Texas, USA). Thymidine (T9250), IdU (5-lodo-2′-deoxyuridine) (I7125), and CldU (5-chloro-2′-deoxyuridine) (C6891) were purchased from Sigma–Aldrich (St. Louis, MO, USA). EdU (Ab219801) was purchased from Abcam (Cambridge, UK). MTS (BB-4204) was purchased from Bestbio (Nanjing, China). The following primary antibodies were used from the following manufacturers: rabbit anti-NAT10 (Abcam Cat # Ab194297), rabbit anti-CDC6 (Abcam Cat # Ab188423), rabbit anti-AR (Abcam Cat #Ab108341), rabbit anti-Lamin B1 (Abcam Cat # Ab16048), rat anti-BrdU (Abcam Cat # Ab6326), mouse anti-β-actin (Origene Cat # TA347340), mouse anti-BrdU (Bioworld Cat # MB6004), and rabbit anti-PCNA (Bioworld Cat # BS1289). The following secondary antibodies were purchased from the following manufacturers: anti-rat Alexa Fluor 488 (Invitrogen Cat # A21470) and anti-IgG Alexa Fluor 488- or tetramethyl rhodamine isothiocyanate (TRITC)-conjugated (Origene, Rockville, MD, USA).

### 4.2. Cell Culture

Prostate cancer cells (PC3, DU145, VCaP, and LNCaP) were purchased from the National Infrastructure of Cell Line Resource. Cells were maintained in Roswell Park Memorial Institute (RPMI) 1640 Medium supplemented with 10% fetal bovine serum (Gibco, Life Technologies, Carlsbad, CA, USA). Cells were incubated in a humidified atmosphere with 5% CO_2_ at 37 °C. Cells were regularly passaged and checked for mycoplasma contamination.

### 4.3. Cell Proliferation Assay

Cell proliferation was assessed using MTS (BestBio, Beijing, China). Briefly, 3000 cancer cells were seeded into 96-well plates, cultured or treated at the indicated times, and incubated with MTS solution (10 μL/well) for 2 h at 37 °C. Absorbance was measured at 490 nm using a microplate reader (Multiskan Go, Thermo Scientific, Waltham, MA, USA). All experiments were performed in quadruplicate.

### 4.4. Colony Formation Assay

Colony formation assays were performed as previously described [37]. Briefly, cells in the logarithmic growth phase were seeded into 6-well plates at a density of 800 cells/well, and allowed to grow for 12 h, followed by treatment with Remodelin (0, 2, 10, 20, or 40 μM) for 14 days. The cells were subsequently fixed in 4% paraformaldehyde for 15 min at room temperature, and stained with 0.1% crystal violet for 10 min. The total number of colonies (>50 cells/colony) was manually counted and imaged using a microscope. The rate of colony formation was calculated using the following equation: colony-formation rate = (number of colonies/number of cells incubated) × 100%. The experiment was repeated three times.

### 4.5. Wound Healing Assay

A total of 1 × 10^5^ cells were cultured in 6-well plates with complete medium. When the cells reached 90% confluence, a wound was created in the cell monolayer using a 100 μL micropipette tip, and the cells were then washed with phosphate-buffered saline (PBS) to remove any debris from the wound. After culturing in serum-free medium (with or without Remodelin) for 24 h at 37 °C, the cells that had migrated into the scratched region were evaluated and imaged using an inverted microscope (Olympus, Tokyo, Japan). Images were analyzed using ImageJ software, and the experiment was repeated three times.

### 4.6. Cell Migration and Invasion Assays

Cell migration and invasion were assessed using a Transwell assay with a pore size of 8 μm (Millipore, Bedford, MA, USA). In brief, cells were washed twice with PBS and resuspended in serum-free medium, and the cell density was adjusted to 1 × 10^5^. Then, 200 μL of cell suspension was added to the upper compartment of a 24-well Transwell culture chamber. The lower compartment was filled with 600 μL of complete medium. After incubation for 24 h at 37 °C, the cells that had failed to traverse into the upper compartment were removed using a wet cotton swab. Traversed cells on the lower side of the filter were then fixed in methanol for 30 min (Sigma–Aldrich). Then, these cells were stained with 0.5% crystal violet (Merck, Darmstadt, Germany) for 20 min and counted using a microscope (Olympus, Tokyo, Japan). The method of cell invasion was similar to that of cell migration, except that the inserts were coated with BD Matrigel Matrix (BD Biosciences, New York, NY, USA). Images were analyzed using ImageJ software, and the experiment was repeated three times.

### 4.7. Mouse Transplantation Experiment

All procedures were approved by the Peking University Institutional Animal Use and Care Committee. Four-week-old male nude athymic BALB/c nu/nu mice (9 in total) were purchased for the experiment. PC-3 cells (6 × 10^6^) were resuspended in PBS solution and inoculated into the armpits of mice by subcutaneous injection. One week later, Remodelin (low-dose: 2 mg/kg; high-dose: 20 mg/kg) injection was performed every two days for a total of 4 weeks. Tumor size was measured every two days and calculated using the following formula: [length (mm) × width^2^ (mm^2^) × 0.5]. Tumors were excised from nude mice and weighed after 4 weeks. The xenograft tumor tissue samples were fixed in 4% neutral formalin, embedded in paraffin, and then sectioned for HE and immunohistochemical staining. The common drug injection for euthanasia includes a sodium pentobarbital injection. When mice are injected intraperitoneally with sodium pentobarbital at 150 mg/kg, they stop breathing. In order to ensure that the mice had been euthanized, the animals’ heart beats were checked if necessary.

### 4.8. Hematoxylin and Eosin (HE) Staining and Immunohistochemistry

HE staining and immunohistochemistry were performed as previously described [15]. Briefly, sections (4 μm thick) from tissues were dewaxed, rehydrated, and subjected to hematoxylin and eosin staining or immunohistochemistry, respectively. After antigen retrieval and blocking with 3% hydrogen peroxide, the sections were incubated with 10% goat serum for 30 min at room temperature, and then incubated with rabbit anti-NAT10 polyclonal antibody at 4 °C overnight. Color development was performed by using 3,3-diaminobezidine (DAKO, Carpinteria, CA, USA). NAT10 (1:500; Abcam) was considered to be positive in the cytoplasm and nucleus.

### 4.9. Western Blotting

Total cell lysates were obtained by incubating the cells in 0.5% NP40 lysis buffer for 30 min on ice. After centrifugation at 10,000× *g* for 10 min at 4 °C, the supernatant was collected and stored at −20 °C for subsequent analysis. Western blotting was performed as previously described [38]. Equal amounts of cell proteins (20–40 μg/lane) were separated by SDS-PAGE in 10% gels and transferred to PVDF membranes (Millipore, Billerica, MA, USA) using a semidry transfer cell (Bio-Rad, Hercules, CA, USA) at 25 V for 40 min. The membranes were then blocked for 1 h with TBS-T (20 mmol/L Tris-HCl pH 7.6, 137 mmol/L NaCl and 0.1% Tween-20) containing 5% nonfat dry milk (BD Biosciences), or with 1% BSA (Sigma Aldrich, St. Louis, MO, USA), and incubated overnight with primary antibodies. After the membranes were washed, they were incubated for 1 h with peroxidase-conjugated goat anti-rabbit IgG, or peroxidase-conjugated goat anti-mouse IgG. The proteins were visualized using an enhanced chemiluminescence kit (Bio-Rad, Hercules, CA, USA). Band images of three independent experiments were quantified by optical density using Lab-Works 4.6 software (Bio-Rad, Hercules, CA, USA). β-actin was used as an internal control for each protein. The antibodies included anti-NAT10 (1:2000), anti-CDC6 (1:1000), anti-AR (1:200), and peroxidase-conjugated goat anti-rabbit and goat anti-mouse IgG (Origene).

### 4.10. Immunofluorescence

Immunofluorescence was performed as previously described [39]. Cells were seeded on coverslips at a density of 1 × 10^5^ cells/mL in 24-well microplates and grown overnight in complete medium. Coverslips were rinsed with PBS once and incubated in a 4% paraformaldehyde solution for 15 min at room temperature, followed by three PBS washes. Cells were permeabilized with PBS containing 0.2% Triton X-100 for 10 min at room temperature, followed by three PBS washes. Cell were blocked in PBS containing 5% donkey serum for 1 h at room temperature. Incubation of anti-Lamin B1 (1:300; Santa Cruz Biotechnology, Dallas, TX, USA) and anti-NAT10 (1:2000; Abcam) was performed at 4 °C overnight, and after several washes, Alexa Fluor 488- or tetramethylrhodamine isothiocyanate (TRITC)-conjugated IgG secondary antibodies (Origene) were added for 1 h. Nuclei were counterstained with DAPI (Origene) for 10 min. Images were acquired on a fluorescence microscope (Model CX51; Olympus, Tokyo, Japan), and Photoshop version 7.0 (Adobe Systems Inc., San Jose, CA, USA) was used to analyze the results.

### 4.11. 5-Ethynyl-2′-deoxyuridine (EdU) Analysis

Cell proliferation was measured using an EdU kit purchased from Abcam (Shanghai, China). Cells were seeded on coverslips at a density of 1 × 10^5^ cells/mL in 24-well microplates, and grown overnight in complete medium. EdU (10 μM) was added and culture was continued for 2 h. Coverslips were rinsed with PBS once and incubated in a 4% paraformaldehyde solution for 15 min at room temperature, followed by three PBS washes. Cells were permeabilized with PBS containing 0.2% Triton X-100, for 10 min at room temperature, followed by three PBS washes. Then, the EdU click reaction (TBS 430 μL, CuSO_4_ 20 μL, iFlour 488 Azide 1.2 μL, and EdU additive solution 50 μL) was added for 30 min at room temperature (avoid light) followed by three PBS washes. Cells were stained with Hoechst for 10 min at room temperature and sealed with the coverslips. After performing all of the steps in the manufacturer’s instructions, the samples were visualized using a fluorescence microscope (Olympus, Tokyo, Japan).

### 4.12. IdU-CldU In Situ Labeling Assay

The cultured cells were incubated with IdU (20 μM) for 20 min and then labeled with CldU (200 μM) for 1 h. Remodelin (20 μM) treatment was optional and could be performed before or after IdU labeling. Cells were extracted with 0.5% Triton X-100 containing CSK buffer (10 mM MOPS, 100 mM NaCl, 300 mM sucrose, and 3 mM MgCl_2_) at room temperature for 10 min and fixed in 4% paraformaldehyde (diluted in CSK buffer) on ice for 30 min. The cells were treated with 2.5 M hydrochloric acid at room temperature for 1 h, and neutralized using 0.1 M borate buffer. After blocking with 3% BSA at room temperature for 1 h, the cells were incubated in the presence of anti-BrdU (ab6326, Abcam) and anti-BrdU (MB6004, Bioworld) at room temperature for 1 h, and then with secondary antibodies conjugated to Alexa Fluor 488 or tetramethylrhodamine isothiocyanate (TRITC) (Origene). A fluorescence microscope (Olympus) was used to observe and image the samples.

### 4.13. DNA Fiber Assay

DNA fiber assays were performed as previously described [40]. Cells were incubated with IdU (20 μM) for 20 min, followed by CldU (200 μM) mixed with Remodelin (20 μM) or DMSO for 20 min. In some experiments, 4 mM hydroxyurea (HU) was added at 37 °C for 4 h. After trypsin digestion, the cells were placed on ice, counted, and diluted to 5 × 10^5^/mL in PBS. The labeled cells were diluted with unlabeled cells (1:5), and then 2.5 μL was added onto slides and lysed in lysis solution (200 mM Tris-HCl, 50 mM EDTA, and 0.5% SDS) for 10 min. The slides were tilted 30° and the droplets flowed slowly down at a constant speed. After drying completely, the slides were fixed in methanol/acetic acid (3:1) for 10 min, denatured in 2.5 M hydrochloric acid for 1 h, and neutralized with 0.1 M borate buffer. After blocking in 5% BSA at 37 °C for 30 min, anti-BrdU (ab6326, Abcam) and anti-BrdU (MB6004, Bioworld, Irving, TX, USA) was added to the slides at 37 °C for 1.5 h. After PBST cleaning, slides were incubated with Alexa Fluor 488- or tetramethyl rhodamine isothiocyanate (TRITC)-conjugated secondary antibodies (Origene) at 37 °C for 45 min. Fluorescence microscopy (Olympus, Tokyo, Japan) was used to observe and image the slides.

### 4.14. iPOND Assay

VCaP cells (a total of 1 × 10^8^ cells) were labeled with 15 μM EdU for 15 min (with or without 10 μM thymidine chase). After EdU labeling, all samples were fixed in 1% formaldehyde for 20 min at room temperature, followed by a 5 min incubation with 0.125 M glycine to quench the formaldehyde. Fixed samples were collected into six 50 mL conical tubes, centrifuged at 900× *g* at 4 °C for 5 min, and washed three times with 0.1% BSA in PBS. Then, the cells were resuspended in 0.1% Triton X-100 in PBS for permeabilization. Pellets were washed once with 0.5% BSA in PBS and once with PBS prior to the click reaction. Briefly, click chemistry reactions were performed to conjugate biotin to EdU-labeled DNA at room temperature for 1 h. Streptavidin beads were used to capture the biotin-conjugated DNA-protein complexes at 4 °C for 16 h. The captured complexes were extensively washed using SDS and high-salt wash buffers. Purified replication fork proteins were eluted under reducing conditions by boiling in 2× SB sample buffer for 25 min. iPOND samples were separated using SDS-PAGE. Anti-PCNA (1:1000) was used as an internal control.

### 4.15. TUNEL Assay

TUNEL Assay (chromogenic method) were performed using a TUNEL Cell Apoptosis Detection Kit (Beyotime Biotechnology, Shanghai, China, C1098). Briefly, the adherent cells were fixed using 4% formaldehyde in PBS for 30 min. The slides were rinsed with PBS, then PBS containing 0.5% Triton X-100 was added; the slides were incubated at room temperature for 5 min. The slides were rinsed with PBS, PBS containing 0.3% H_2_O_2_ was added, and the slides were incubated at room temperature for 20 min. The slides were rinsed with PBS and 50 μL of TdT reaction buffer (TdT reaction buffer containing 0.5 U/μL of TdT enzyme and 40 pmol/μL of biotinylated-dUTP) was pipetted onto the slides, with enough volume to cover the cells. The slides were incubated in a humidified chamber for 60 min at 37 °C. The slides were rinsed with PBS, 0.2 mL labeled reaction termination solution was added, and the slides were incubated at room temperature for 10 min. The slides were rinsed with PBS, 50 μL Streptavidin-HRP buffer (Streptavidin-HRP buffer containing 1 μL Streptavidin-HRP and 49 μL Streptavidin-HRP diluent) was added, and the slides were incubated at room temperature for 30 min. The slides were rinsed with PBS, and 0.2 mL DAB chromogenic solution (the DAB chromogenic solution includes a 1:1 mixture of solution A and solution B) was added. The slides were incubated at room temperature for 10 min. The slides were rinsed with PBS, air dried, and sealed. Light microscopy (Olympus, Tokyo, Japan) was used to observe and image the slides.

### 4.16. Co-immunoprecipitation

The interaction between NAT10 and CDC6 was examined using the standard co-immunoprecipitation (Co-IP) protocol. The cells were harvested using cell scrapers and pelleted by centrifugation at 1500× *g* for 5 min at 4 °C. Ice-cold lysis buffer was added to the cell pellet, followed by incubation for 30 min on ice with vigorous vortexing every 10 min. The samples were then centrifuged at 15,000× *g* for 15 min at 4 °C. The supernatant was transferred to a new tube, 20 μL of protein A/G agarose (Santa Cruz Biotech) was added, and the tube was gently shaken for 10 min at 4 °C. The samples were centrifuged at 3000× *g* for 15 min at 4 °C. The IP antibody was added to the supernatant and then incubated overnight at 4 °C on a tube rotator. The samples were centrifuged at 3000× *g* for 5 min at 4 °C, and the supernatant was removed. The pelleted resin was resuspended in 1 mL of ice-cold washing buffer and incubated for 20 min at 4 °C on a tube rotator. The samples were centrifuged at 3000× *g* for 5 min at 4 °C, and the supernatant was removed again. The washing steps were repeated 3 times. The resin-bound immune complexes were resuspended in 40 μL of 2× SB buffer and then boiled for 15 min. The samples were centrifuged at 3000× *g* for 5 min, and then the supernatant was transferred to a new tube for immunoblotting analysis. Anti-DDDDK-tagged pAb agarose (MBL, Ottawa, IL, USA), anti-GFP mAb-agarose (MBL), anti-IgG (Cell Signaling, Danvers, MA, USA), anti-Flag (1:1000; Origene), and anti-GFP (1:2000; Immunoway, Plano, TX, USA) were used for immuno-coprecipitation.

### 4.17. Chromatin Immunoprecipitation

Chromatin immunoprecipitation (ChIP) assays were performed using a SimpleChIP Plus Sonication Chromatin IP Kit (Cell Signaling Technology). Briefly, VCaP and PC3 (1 × 10^7^) cells were treated with 1% formaldehyde and neutralized by the addition of 125 mM glycine. Cells were washed twice in ice-cold phosphate-buffered saline and lysed in sodium dodecyl sulfate lysis buffer (1% sodium dodecyl sulfate, 10 mM EDTA, and 50 mM Tris-HCl, pH 8.0) containing protease inhibitors, and DNA in the cross-linked chromatin preparations was sonicated to an average fragment size of 0.5 kb. Each sample was incubated overnight at 4 °C, with 5 μg of the antibody for NAT10, CDC6, or the control IgG. After formaldehyde reversal, phenol/chloroform extraction, and ethanol precipitation, the DNA was dissolved in Tris/EDTA buffer. Interaction with predicted promoters was assessed using qPCR with specific primers for LMNB2 and MCM4 [41].

### 4.18. Quantitative Real-Time PCR

SYBR^®^ Premix Ex TaqTM II (Takara, Kyoto, Japan) was used according to the manufacturer’s instructions. All PCRs were performed using an iCycler iQ real-time PCR detection system (Bio–Rad). Specific primers were used, and the annealing temperature was optimized for each primer [42]. For each reaction, the cycling parameters were set as follows: initial denaturation at 95 °C for 5 min; denaturation at 95 °C for 30 s, annealing temperature for 30 s, and 72 °C for 30 s; 35 cycles at 95 °C for 30 s, annealing temperature for 30 s, and 72 °C for 30 s; and final extension at 72 °C for 5 min. The calculation of IP efficiency was performed using the following equation: Percentage Input = 2% × 2 ^(C^[T] ^2% input sample − C^[T] ^IP sample)^, C[T] = C_T_ = Threshold cycle of PCR. The PCR primers used are as follows:

LMNB2 Origin F 5′-GGCTGGGCATGGACTTTCATTTCAG-3′

LMNB2 Origin R 5′-GTGGAGGGATCTTTCTTAGACATC-3′

LMNB2 Distal F 5′- GTTAACAGTCAGGCGCATGGGCC-3′

LMNB2 Distal R 5′- CCATCAGGGTCACCTCTGGTTCC-3′

MCM4 Origin F 5′-GGACATTACAGATGCATTTCTC-3′

MCM4 Origin R 5′-AAGAGTTCCAAGTTTGTTCCTC-3′

MCM4 Distal F 5′-TACCTGTGGGTAAGAGATGAGTTG-3′

MCM4 Distal R 5′-CTCTATACATGCAACGACTTGGG-3′

### 4.19. TCGA Analysis

The analysis on the PRAD (prostate adenocarcinoma) data set from the TCGA database was conducted using the UALCAN website [43]. By entering the genes of NAT10, CDC6, MCM7, and MKI67 in UALCAN, a total of 497 cases of prostate adenocarcinoma with full expression profiles, and pathological and clinical data were analyzed, while the remaining 3 cases without available data were declined. The analysis of gene expression level is based on the RNA-seq data, and 497 cases with RNA-seq data were analyzed for the level of gene expression. The expression levels of NAT10, CDC6, MCM7, and MKI67 were categorized as boxplots based on TPM (transcript per million) analyzing normal vs. tumor, node metastatic status, and the Gleason score group, respectively. The relationships between NAT10 expression and cell proliferation-associated proteins (MCM7, CDC6, and MKI67) were statistically analyzed according to the TPM values. The correlation between NAT10, CDC6, MCM7, and MKI67 expression with the overall survival (OS) duration in PRAD patients were used for Kaplan–Meier survival analyses, and consequently, overall survival plots were generated (patients were classified into high and low/medium expression groups).

### 4.20. Statistical Analysis

All statistical analyses were performed using SPSS (Version 17.0, Chicago), Microsoft Excel 2007, and GraphPad Prism software. For the data in all cellular experiments, an unpaired *t*-test was used for the comparison of two groups. The experiments were independently repeated three times. Statistical comparisons between multiple groups were analyzed using a one-way ANOVA with Tukey’s post-hoc test to correct for multiple testing. The survival curves of the samples with high gene expression and low/medium gene expression were compared by the log rank test. Data are expressed as mean ± SD. All statistical tests were two-sided, and significance was considered as *, *p* < 0.05; **, *p* < 0.01; ***, *p* < 0.001; ****, *p* < 0.0001 and ns, no significance.

## 5. Conclusions

Our data demonstrated that Remodelin, an inhibitor of NAT10, effectively inhibits the proliferation, migration, and invasion of PCa cells in both AR-positive and AR-negative prostate cancer cells. The mechanism of action likely relies on the direct participation of NAT10 in the initiation of DNA replication with the interaction of CDC6. The expression of NAT10 is influenced by AR modulators, indicating its involvement in the development and progression of CRPC. However, the fact that Remodelin still effectively inhibits the growth of castration-resistant prostate cancer cells suggests that targeting NAT10 is a potential strategy for combating CRPC.

## Figures and Tables

**Table 1:** Correlations between the expression of NAT10, CDC6, MCM7, or Ki67, and the clinicopathological features of prostate cancers (*T*-test; *, *p* < 0.05; **, *p* < 0.01; ***, *p* < 0.001).

Comparison	NAT10	CDC6	MCM7	Ki67
**Normal-vs-Tumor**	9.36 × 10^−2^	2.90 × 10^−6^ (***)	3.34 × 10^−8^ (***)	2.07 × 10^−9^ (***)
Normal vs. Gleason score				
**Normal-vs-Gleason score 6**	3.55 × 10^−1^	5.42 × 10^−1^	1.43 × 10^−2^ (*)	1.49 × 10^−1^
**Normal-vs-Gleason score 7**	2.44 × 10^−1^	1.09 × 10^−2^ (*)	4.22 × 10^−3^ (**)	1.17 × 10^−4^ (***)
**Normal-vs-Gleason score 8**	2.51 × 10^−2^ (**)	2.76 × 10^−3^ (**)	5.01 × 10^−7^ (***)	2.29 × 10^−5^ (***)
**Normal-vs-Gleason score 9**	9.64 × 10^−3^ (***)	2.26 × 10^−7^ (***)	6.16 × 10^−11^ (***)	9.07 × 10^−13^ (***)
**Normal-vs-Gleason score 10**	5.01 × 10^−1^	8.51 × 10^−2^	1.89 × 10^−1^	1.09 × 10^−1^
Node metastasis				
**N** **ormal-vs-N0**	7.01 × 10^−2^	5.76 × 10^−5^ (***)	3.16 × 10^−7^ (***)	2.80 × 10^−8^ (***)
**N** **ormal-vs-N1**	3.78 × 10^−3^ (**)	1.45 × 10^−4^ (***)	7.03 × 10^−7^ (***)	3.93 × 10^−9^ (***)
**N** **0-vs-N1**	4.52 × 10^−2^ (*)	2.15 × 10^−2^ (*)	6.16 × 10^−3^ (**)	2.44 × 10^−3^ (**)

## Data Availability

The datasets included in the current study can be obtained from the corresponding author upon reasonable request.

## References

[1] Siegel R.L., Miller K.D., Jemal A. (2019). Cancer statistics, 2019. CA Cancer J. Clin..

[2] Davies A., Conteduca V., Zoubeidi A., Beltran H. (2019). Biological Evolution of Castration-resistant Prostate Cancer. Eur. Urol. Focus.

[3] Mediwala S.N., Sun H., Szafran A.T., Hartig S.M., Sonpavde G., Hayes T.G., Thiagarajan P., Mancini M.A., Marcelli M. (2013). The activity of the androgen receptor variant AR-V7 is regulated by FOXO1 in a PTEN-PI3K-AKT-dependent way. Prostate.

[4] Yan Y.Q., Huang H.J. (2019). Interplay Among PI3K/AKT, PTEN/FOXO and AR Signaling in Prostate Cancer. Adv. Exp. Med. Biol..

[5] Gaillard H., Garcia-Muse T., Aguilera A. (2015). Replication stress and cancer. Nat. Rev. Cancer.

[6] Macheret M., Halazonetis T.D. (2015). DNA Replication Stress as a Hallmark of Cancer. Annu. Rev. Pathol. Mech. Dis..

[7] Arango D., Sturgill D., Alhusaini N., Dillman A.A., Sweet T.J., Hanson G., Hosogane M., Sinclair W.R., Nanan K.K., Mandler M.D. (2018). Acetylation of Cytidine in mRNA Promotes Translation Efficiency. Cell.

[8] Ito S., Horikawa S., Suzuki T., Kawauchi H., Tanaka Y., Suzuki T., Suzuki T. (2014). Human NAT10 is an ATP-dependent RNA acetyltransferase responsible for N4-acetylcytidine formation in 18 S ribosomal RNA (rRNA). J. Biol. Chem..

[9] Lv J., Liu H., Wang Q., Tang Z., Hou L., Zhang B. (2003). Molecular cloning of a novel human gene encoding histone acetyltransferase-like protein involved in transcriptional activation of hTERT. Biochem. Biophys. Res. Commun..

[10] Liu H., Ling Y., Gong Y., Sun Y., Hou L., Zhang B. (2007). DNA damage induces *N*-acetyltransferase NAT10 gene expression through transcriptional activation. Mol. Cell. Biochem..

[11] Shen Q., Zheng X., McNutt M.A., Guang L., Sun Y., Wang J., Gong Y., Hou L., Zhang B. (2009). NAT10, a nucleolar protein, localizes to the midbody and regulates cytokinesis and acetylation of microtubules. Exp. Cell Res..

[12] Larrieu D., Britton S., Demir M., Rodriguez R., Jackson S.P. (2014). Chemical Inhibition of NAT10 Corrects Defects of Laminopathic Cells. Science.

[13] Oh T.I., Lee Y.M., Lim B.O., Lim J.H. (2017). Inhibition of NAT10 Suppresses Melanogenesis and Melanoma Growth by Attenuating Microphthalmia-Associated Transcription Factor (MITF) Expression. Int. J. Mol. Sci..

[14] Dalhat M.H., Mohammed M.R.S., Ahmad A., Khan M.I., Choudhry H. (2021). Remodelin, a N-acetyltransferase 10 (NAT10) inhibitor, alters mitochondrial lipid metabolism in cancer cells. J. Cell. Biochem..

[15] Cao Y., Yao M., Wu Y., Ma N., Liu H., Zhang B. (2020). N-Acetyltransferase 10 Promotes Micronuclei Formation to Activate the Senescence-Associated Secretory Phenotype Machinery in Colorectal Cancer Cells. Transl. Oncol..

[16] Akerblom L., Ehrenberg A., Graslund A., Lankinen H., Reichard P., Thelander L. (1981). Overproduction of the free radical of ribonucleotide reductase in hydroxyurea-resistant mouse fibroblast 3T6 cells. Proc. Natl. Acad. Sci. USA.

[17] Reichard P., Ehrenberg A. (1983). Ribonucleotide reductase—A radical enzyme. Science.

[18] Dungrawala H., Cortez D. (2015). Purification of proteins on newly synthesized DNA using iPOND. Methods Mol. Biol..

[19] Warbrick E. (2000). The puzzle of PCNA’s many partners. Bioessays.

[20] Bai V.U., Cifuentes E., Menon M., Barrack E.R., Reddy G.P. (2005). Androgen receptor regulates Cdc6 in synchronized LNCaP cells progressing from G1 to S phase. J. Cell. Physiol..

[21] Jin F., Fondell J.D. (2009). A novel androgen receptor-binding element modulates Cdc6 transcription in prostate cancer cells during cell-cycle progression. Nucleic Acids Res..

[22] Karanika S., Karantanos T., Li L., Wang J., Park S., Yang G., Zuo X., Song J.H., Maity S.N., Manyam G.C. (2017). Targeting DNA Damage Response in Prostate Cancer by Inhibiting Androgen Receptor-CDC6-ATR-Chk1 Signaling. Cell Rep..

[23] Liu Y., Gong Z., Sun L., Li X. (2014). FOXM1 and androgen receptor co-regulate CDC6 gene transcription and DNA replication in prostate cancer cells. Biochim. Biophys. Acta.

[24] Duncker B.P., Chesnokov I.N., McConkey B.J. (2009). The origin recognition complex protein family. Genome Biol..

[25] Litvinov I.V., Vander Griend D.J., Antony L., Dalrymple S., De Marzo A.M., Drake C.G., Isaacs J.T. (2006). Androgen receptor as a licensing factor for DNA replication in androgen-sensitive prostate cancer cells. Proc. Natl. Acad. Sci. USA.

[26] Murthy S., Wu M., Bai V.U., Hou Z.Z., Menon M., Barrack E.R., Kim S.H., Reddy G.P.V. (2013). Role of Androgen Receptor in Progression of LNCaP Prostate Cancer Cells from G(1) to S Phase. PLoS ONE.

[27] Shi Y.K., Yu Y.P., Zhu Z.H., Han Y.C., Ren B., Nelson J.B., Luo J.H. (2008). MCM7 interacts with androgen receptor. Am. J. Pathol..

[28] Yoshida K., Sugimoto N., Iwahori S., Yugawa T., Narisawa-Saito M., Kiyono T., Fujita M. (2010). CDC6 interaction with ATR regulates activation of a replication checkpoint in higher eukaryotic cells. J. Cell. Sci..

[29] Antonarakis E.S., Lu C., Wang H., Luber B., Nakazawa M., Roeser J.C., Chen Y., Mohammad T.A., Chen Y., Fedor H.L. (2014). AR-V7 and resistance to enzalutamide and abiraterone in prostate cancer. N. Engl. J. Med..

[30] Joseph J.D., Lu N., Qian J., Sensintaffar J., Shao G., Brigham D., Moon M., Maneval E.C., Chen I., Darimont B. (2013). A clinically relevant androgen receptor mutation confers resistance to second-generation antiandrogens enzalutamide and ARN-509. Cancer Discov..

[31] Harris W.P., Mostaghel E.A., Nelson P.S., Montgomery B. (2009). Androgen deprivation therapy: Progress in understanding mechanisms of resistance and optimizing androgen depletion. Nat. Clin. Pract. Urol..

[32] Arora V.K., Schenkein E., Murali R., Subudhi S.K., Wongvipat J., Balbas M.D., Shah N., Cai L., Efstathiou E., Logothetis C. (2013). Glucocorticoid receptor confers resistance to antiandrogens by bypassing androgen receptor blockade. Cell.

[33] Mu P., Zhang Z., Benelli M., Karthaus W.R., Hoover E., Chen C.C., Wongvipat J., Ku S.Y., Gao D., Cao Z. (2017). SOX2 promotes lineage plasticity and antiandrogen resistance in TP53- and RB1-deficient prostate cancer. Science.

[34] Markowski M.C., Chen Y., Feng Z., Cullen J., Trock B.J., Suzman D., Antonarakis E.S., Paller C.J., Rosner I., Han M. (2019). PSA Doubling Time and Absolute PSA Predict Metastasis-free Survival in Men With Biochemically Recurrent Prostate Cancer After Radical Prostatectomy. Clin. Genitourin Cancer.

[35] Wu Y., Cao Y., Liu H., Yao M., Ma N., Zhang B. (2020). Remodelin, an inhibitor of NAT10, could suppress hypoxia-induced or constitutional expression of HIFs in cells. Mol. Cell. Biochem..

[36] Zhang H., Hou W., Wang H.L., Liu H.J., Jia X.Y., Zheng X.Z., Zou Y.X., Li X., Hou L., McNutt M.A. (2014). GSK-3 beta-Regulated N-Acetyltransferase 10 Is Involved in Colorectal Cancer Invasion. Clin. Cancer Res..

[37] Yang Z., Zhang Q., Luo H., Shao L., Liu R., Kong Y., Zhao X., Geng Y., Li C., Wang X. (2020). Effect of Carbon Ion Radiation Induces Bystander Effect on Metastasis of A549 Cells and Metabonomic Correlation Analysis. Front. Oncol..

[38] Kurien B.T., Scofield R.H. (2015). Western blotting: An introduction. Methods Mol. Biol..

[39] Odell I.D., Cook D. (2013). Immunofluorescence techniques. J. Investig. Dermatol..

[40] Quinet A., Carvajal-Maldonado D., Lemacon D., Vindigni A. (2017). DNA Fiber Analysis: Mind the Gap!. Methods Enzymol..

[41] Sugimoto N., Maehara K., Yoshida K., Yasukouchi S., Osano S., Watanabe S., Aizawa M., Yugawa T., Kiyono T., Kurumizaka H. (2015). Cdt1-binding protein GRWD1 is a novel histone-binding protein that facilitates MCM loading through its influence on chromatin architecture. Nucleic Acids Res..

[42] Sugimoto N., Yugawa T., Iizuka M., Kiyono T., Fujita M. (2011). Chromatin Remodeler Sucrose Nonfermenting 2 Homolog (SNF2H) Is Recruited onto DNA Replication Origins through Interaction with Cdc10 Protein-dependent Transcript 1 (Cdt1) and Promotes Pre-replication Complex Formation. J. Biol. Chem..

[43] Chandrashekar D.S., Bashel B., Balasubramanya S.A.H., Creighton C.J., Ponce-Rodriguez I., Chakravarthi B., Varambally S. (2017). UALCAN: A Portal for Facilitating Tumor Subgroup Gene Expression and Survival Analyses. Neoplasia.

